# Dr. Chase and His Theories

**Published:** 1877-08

**Authors:** J. Campbell


### ARTICLE II.

### *Dr. Chase and his Theories.*

BY J. CAMPBELL.

The dental profession should certainly feel itself under obligations to Dr. Chase for the persistency with which he has pursued the investigation of Oral Electricity, and the elaborate manner in which he has treated the subject, both in the June number of the *Dental Journal* for 1876, as well as in his article in its issue of last month. In saying this much, however, I do not wish to be understood as claiming that the dental profession should adopt his theories so far as they lead to a radical change in the material for filling teeth. I simply desire to recognize in Dr. Chase's course an example of intelligent scientific research, which it would be well for the profession to imitate.

His conclusions may be wrong, in fact it would seem that the weight of evidence is against him, if the experience of half a century is worth anything; but this I hold does not detract from the credit we should accord him for his indefatigable labor and patient searching after truth.

It will be a difficult matter to refute the conclusions reached by Dr. Chase, providing we admit that the same laws lead to the disintegration of animate substances that operate in the dissolution of the inanimate.

But we have no means of ascertaining that such is the case, but on the contrary we have reasonable grounds for concluding that such is not the case. In the absence of experiments having all conditions alike, being possible, this however must remain a matter of partial conjecture.

Were it possible to determine with the accuracy of analysis what medicines would correct a derangement of the living tissue, then medicine would no longer be an art, but a science, whose claims would rest on the same foundation with that of chemistry or mathematics.

But science can go no farther than to take cognizance of physical principles minus the highest of all principles—LIFE.

In concluding an analysis, with the view of applying such results as may be reached to the healing art, we cannot say that the analysis has given us positive grounds for proceeding.

We may determine what monads are, what proximate principles are, what tissue is; but we cannot determine what an organ is, any further than to know that it performs certain functions. We may analyze their material elements, that is, separate them, but we cannot put them together so as to make an organ.

In the progress of the healing art it cannot be said that everything is due to analysis, or the application of scientific principles. The knowledge of the destructive properties of many poisons was obtained by experiments made on the living tissue. On dead tissue many of them are wonderfully potent as conservators, and hence if analogy were followed would be also conservators of living tissue. But on living tissue they are just the reverse. It may be stated here that medicine for the most part is dependent on empyrical knowledge rather than on scientific knowledge, that is the physician does not conclude that because this or that substance is composed of certain chemical elements, it therefore must necessarily produce certain results on the living organism ; but he determines the potency of his medicine by the absolute result that it *does* produce. If you ask him to account for such results scientifically he will answer that he cannot. Scientific experiments that are made with the view of applying them to the healing art are defective in this, that the chief factor, vital force, is left out.

What reliability we can place on an experiment where the chief factor is missing, I will leave the scientific world to determine.

The experiments made by Dr. Chase, and the results reached, lead to the overthrow of our heretofore practiced mode of preserving teeth. This would be no great loss to us were we certain that Dr. Chase is right, and that we have been wrong for the whole period of our lives. It is to be hoped that the dental profession is not so wedded to a single mode of practice that proof of its being defective would fail to bring about a modification.

I now propose to examine briefly the nature of the evidence on which we are required to surrender our present mode of practice, together with the nature aud amount of evidence which would permit us to retain it.

The indicative point in favor of a departure is a purely scientific one, and is chiefly based on a class of experiments made by Dr. Chase, showing the galvanic action of metals in the mouth, and their comparative destructive tendency, on the teeth. He tells us that a plug in a tooth, immersed in the fluids of the mouth, is at once a battery ready for action. Gold being the farthest removed from dentos, on the electro-negative scale, " there would be a stronger current than between any other two substances; consequently the dentos would be broken down or dissolved more rapidly than if it would be united to tin, amalgam, or any other material in the list." This result Dr. Chase arrived at after having made repeated tests, and is therefore satisfied to base his practice on it. Now, we are free to confess that, did we lack all the experience in the results of filling teeth, this purely scientific test would have considerable weight with us, even made, as it was, out of the mouth. We might be inclined to pass over the fact that the fluids of the mouth are not always acid, and therefore unable to act as a disintegrative agent, or even when they are acid, and the battery at work, that the work must be done on living tissue, and not on dead bone, as was the case in the experiment. The conclusions reached from this class of evidence would be similar to those reached if we were to saturate a piece of lean meat or muscle with arsenic. We would observe at once that the arsenic preserved the meat from disintegration, and therefore we might conclude that it would also preserve the living tissue from disintegration.

We might be led to forget, in our eagerness to arrive at a conclusion, that we left out of consideration the greatest factor of all, the vital force.

Our experiment, therefore, the moment we put it into practice would fall to the ground. The arsenic, in place of preserving the living tissue, as it did the dead tissue, would destroy it. Our experiment would be worth less as furnishing us a criterion to guide us in determining the effect of arsenic on the living organism, and we would be forced, if we pursued the investigation of the subject, to conclude that arsenic will kill the living, notwithstanding its power of preserving the dead tissue But the arsenic furnishes us with no single exception of what I will call an anomaly in science—and yet it is not an anomaly because the conditions are dissimilar. I might embrace many other articles found in the materia medica of our country, which, were we to follow the indications manifested in a scientific experiment, we would either poison or seriously injure one half the people of the country. My purpose is not so much to elaborate as to show at once that the healing art relies, not so much on purely scientific experiments from which deductions must be drawn, as it does on absolute experiments with the living subject. The character of testimony is by far the most reliable from a medical stand-point, in fact is the only one on which any implicit reliance can be placed.

The question then with us resolves itself into one of absolute results, obtained from a long course of practice—not of results obtained from one man, but from that of hundreds. The weight of this evidence must be placed over against that of the purely scientific experimentalist. If it favor him, so much the stronger will his position be made. If it is against him, then his position will be weakened in proportion to the amount and character of the testimony.

It will be safe to say here that there is not over one first-class operator in fifty, in this country, who has not obtained better results from the use of gold in the mouth than from any other material.

This is their testimony. This is the testimony of two or three generations of dentists. I am not supposing now that any self-interest has given solidity to this concurrent opinion, or that an ignorant prejudice has prevented the majority of our intelligent dentists giving the other materials for filling teeth a fair trial. I am aware that some have testified against the use of amalgam, and when pushed to the wall acknowledge that they never used it. Their testimony is worthless. They belong to a class of men with open mouths and closed heads, whose opinions it would be well to ignore under ordinary circumstances. I count their testimony, or condemnation, as utterly outside the pale of recognition.

But I mean to assert that forty nine out of fifty of our fair-minded men, who are not afraid to go out in search of an opinion of their own, have found more satisfactory results from gold fillings than from amalgam, or any other of the compounds or materials used. This preponderance of testimony—overwhelming in amount and character—

must be placed over against the deductions made from the scientific experiments of Dr. Chase. In this particular Dr. Chase occupies a position similar to the man who, after having made his experiments with arsenic on dead tissue, would insist that it was not destructive to living tissue, while his declarations were made in the face of the protests of forty-nine out of fifty of the medical faculty. He might contend that they could advance no scientific argument against the deathly nature of the arsenic, but on the contrary he could show from analysis that arsenic was not poisonous, or at least that it ought not to be poisonous. In this particular he might gain a seeming advantage in the argument, while his opponents would be compelled to fall back on their experience in lieu of his scientific deductions.

But if forty-nine out of every fifty of the best men in the medical profession would testify that arsenic would kill, that they tried it, and knew from experience, that there could be no mistaking its character in this direction, although being unable to explain why it did kill, we would, in all probability, act on their advice and refrain from swallowing it

Between what, seemingly to the scientific man, ought to be, and what really is, there is very often irreconcilable difficulties. This arises from the fact I have already stated, that the scientist's experiments embrace as factors the physical forces, but do not embrace the vital forces.

His conclusions, therefore, may be partially, or totally defective. They need confirmation by absolute tests on the living organism. Without this we can only regard them in the light of possibilities.

The logic of Dr. Chase's article points in one single direction, and that is, that the electric current is mainly, if not altogether, accountable for the breaking down of a tooth after it is filled. This inference is based, as I have already shown, on a series of experiments conducted with conditions dissimilar to those that must necessarily exist in the mouth. It would be asking too much of the profession to rely with equal faith on the correctness of such views, particularly where it would lead to the total overthrow of a system of practice that has formed the foundation stone of dentistry. We can readily see how the purely scientific experimentalist can sneer at such reasoning as those who rely on experience can advance. But I take the liberty of again reminding such that the great art of medicine relies more on the absolute effects produced than on speculative deductions.

REMARKS BY THE EDITOR, (DR. CHASE.)

I grant that gold has, during the *past*, been superior to all other known substances for the general filling of teeth. Tin was too soft for masticating purposes.

The amalgams of the past were restless—always changing shape—and contracted so as to allow of leakage. *A great change has taken place.* Alloys have been on the market for two or three years, which, poor as most of them are, will better preserve teeth from decay, as a general rule, than the *average* of gold plugs. The best of them *do not* leak. The majority of gold plugs *do leak.*

For myself, since last September I have made an alloy consisting of gold, 20 to 33 per cent.; tin, 40 to 50 per cent.; and silver, 20 to 40 per cent., according to definite, exact, and uniform formulæ. These formulæ I have freely given to the profession.

These gold alloys *do not* leak. They discolor not at all, or very little, in the mouth. In a *large majority of mouths*, not at all. Their plasticity enables one to easily make a water-tight plug in any cavity. It is reasonable to suppose that such an alloy is a thousand times more useful than any alloy previous to three years ago. And even these poor alloys preserved teeth many years that gold foil had condemned to extraction.—*Missouri Dent. Jour.*

